# Health Monitoring Using Smart Home Technologies: Scoping Review

**DOI:** 10.2196/37347

**Published:** 2023-04-13

**Authors:** Plinio P Morita, Kirti Sundar Sahu, Arlene Oetomo

**Affiliations:** 1 School of Public Health Sciences, University of Waterloo Waterloo, ON Canada; 2 Institute of Health Policy, Management, and Evaluation, University of Toronto Toronto, ON Canada; 3 Research Institute of Aging, University of Waterloo Waterloo, ON Canada; 4 Department of Systems Design Engineering, University of Waterloo Waterloo, ON Canada; 5 Centre for Digital Therapeutics, University Health Network Toronto, ON Canada

**Keywords:** monitor, smart home, ambient assisted living, active assisted living, AAL, assisted living, review, internet of things, aging, gerontology, elder, older adult, older people, geriatric, digital health, eHealth, smart technology, older population, independent living, big data, machine learning, algorithm, deep learning

## Abstract

**Background:**

The Internet of Things (IoT) has become integrated into everyday life, with devices becoming permanent fixtures in many homes. As countries face increasing pressure on their health care systems, smart home technologies have the potential to support population health through continuous behavioral monitoring.

**Objective:**

This scoping review aims to provide insight into this evolving field of research by surveying the current technologies and applications for in-home health monitoring.

**Methods:**

Peer-reviewed papers from 2008 to 2021 related to smart home technologies for health care were extracted from 4 databases (PubMed, Scopus, ScienceDirect, and CINAHL); 49 papers met the inclusion criteria and were analyzed.

**Results:**

Most of the studies were from Europe and North America. The largest proportion of the studies were proof of concept or pilot studies. Approximately 78% (38/49) of the studies used real human participants, most of whom were older females. Demographic data were often missing. Nearly 60% (29/49) of the studies reported on the health status of the participants. Results were primarily reported in engineering and technology journals. Almost 62% (30/49) of the studies used passive infrared sensors to report on motion detection where data were primarily binary. There were numerous data analysis, management, and machine learning techniques employed. The primary challenges reported by authors were differentiating between multiple participants in a single space, technology interoperability, and data security and privacy.

**Conclusions:**

This scoping review synthesizes the current state of research on smart home technologies for health care. We were able to identify multiple trends and knowledge gaps—in particular, the lack of collaboration across disciplines. Technological development dominates over the human-centric part of the equation. During the preparation of this scoping review, we noted that the health care research papers lacked a concrete definition of a smart home, and based on the available evidence and the identified gaps, we propose a new definition for a smart home for health care. Smart home technology is growing rapidly, and interdisciplinary approaches will be needed to ensure integration into the health sector.

## Introduction

Smart home technology is rapidly becoming a permanent fixture in our everyday lives. Globally, there are 175 million connected smart homes—a number projected to continue rising. Smart home technology employs the Internet of Things (IoT) concept to interconnect and share data among household devices across a Wi-Fi–based wireless network [1]. Through connection and automated action, smart homes provide convenience and comfort to homeowners [2-4]. Smart devices can include video monitors, motion sensors, alarms, smart planners or calendars, and thermostats. Data can be leveraged for a variety of purposes, including energy saving [5], security and safety [6], fall detection [7], light management [8], and fire detection [7]. However, the benefits of smart home technology run deeper than the superficial hype of comfort and convenience. These may be the solutions to our health care crisis.

The COVID-19 pandemic revealed what many health professionals already suspected: our health care system is overburdened. Our aging population places increased demand on the health care system. Many services are inaccessible to remote communities. Long-term care homes face high mortality and morbidity. To relieve an overwhelmed system, health care is turning to technology [9]—specifically, the application of smart home devices to support independent living. Through continuous behavioral monitoring, IoT devices can be harnessed to detect, diagnose, and monitor health conditions. At the community level, the collection and analysis of sensor data could inform public health initiatives. Interdisciplinary research teams are already working on the application of smart devices in health care. For example, smart wearable trackers, passive infrared sensors, and chair occupancy sensors deliver daily insights into the physical activity levels. Smart thermostats and bed occupancy sensors have been used to track sleep patterns. As physical activity and sleep are good overall health predictors, these can be powerful tools for motivating healthy behavioral changes [10]. The application of machine learning to these systems can be used for behavior change detection [11,12]. Applications can include monitoring the onset and progression of age-related diseases [10], detection of hazardous events (such as falls), and analyzing behavioral impacts following health interventions such as cancer treatments or physical therapy [12]. Information exchange with primary health care providers and caregivers will strengthen health care delivery. Public health authorities could also assess, in real time, the implications of COVID-19 lockdown policies at the population level. These data can be used to inform care delivery, support evidence-based policy making, and enhance care strategies in real time.

The main advantage of using IoT technologies is that they provide objective data in real time. Sensor data are collected passively without human effort; one can go about their day, forgetting about the device. The data are therefore less prone to performance and recall biases compared to the traditional data collection methods. As data are collected continuously and uploaded to the cloud storage, they are immediately available for analysis. The analysis can be conducted automatically, and the resulting insights can be shared immediately with users. The development and deployment of smart home technology for health care will require the concerted effort of an interdisciplinary research team: combining expertise in technology, engineering, and health care. Despite the potential of smart home solutions to health challenges, their real-world implementation continues to be scarce. There is a need to understand the current state of research in smart home technology for health care. Existing reviews on the application of smart home technology in health care are limited [2,3]. Here, we present a scoping review to address this need. The goal was to synthesize the literature on how smart home technologies are being used for health care within the home and community. This study also aims to identify gaps or opportunities in smart home technology to inform practice, policy making, and research. Our review was guided by the following research questions:

1. What smart home technologies are currently being used to monitor health care indicators in vulnerable populations at home or in the community?
2. What types of information are these sensors gathering?
3. What insights can be generated from these data sets?

Our extensive database search led to the identification of 49 peer-reviewed publications on smart home technology for health care, which met our inclusion criteria. We were able to identify multiple research trends and knowledge gaps and provide insight into the next steps needed to propel the field forward.

## Methods

### Data Sources and Search Strategy

This scoping review is based on the widely accepted framework by Arksey and O’Malley [13]. This framework was selected because it allows for the inclusion of various methodological designs across an interdisciplinary field. We searched for papers across 4 databases: PubMed, Scopus, ScienceDirect, and CINAHL. The search terms utilized are presented in Table S1 of Multimedia Appendix 1; they briefly encompassed the following search terms: health, monitor, smart home, ambient assisted living, active assisted living, and AAL. We limited our search to papers published between January 2008 and August 2021. Only peer-reviewed papers published in English were included. Of note, the term “surveillance” was not used in the search query, as its inclusion returned hundreds of results outside of the scope of this research project. A total of 5995 potential papers were identified using the search queries.

### Paper Selection Process

**Figure 1:**
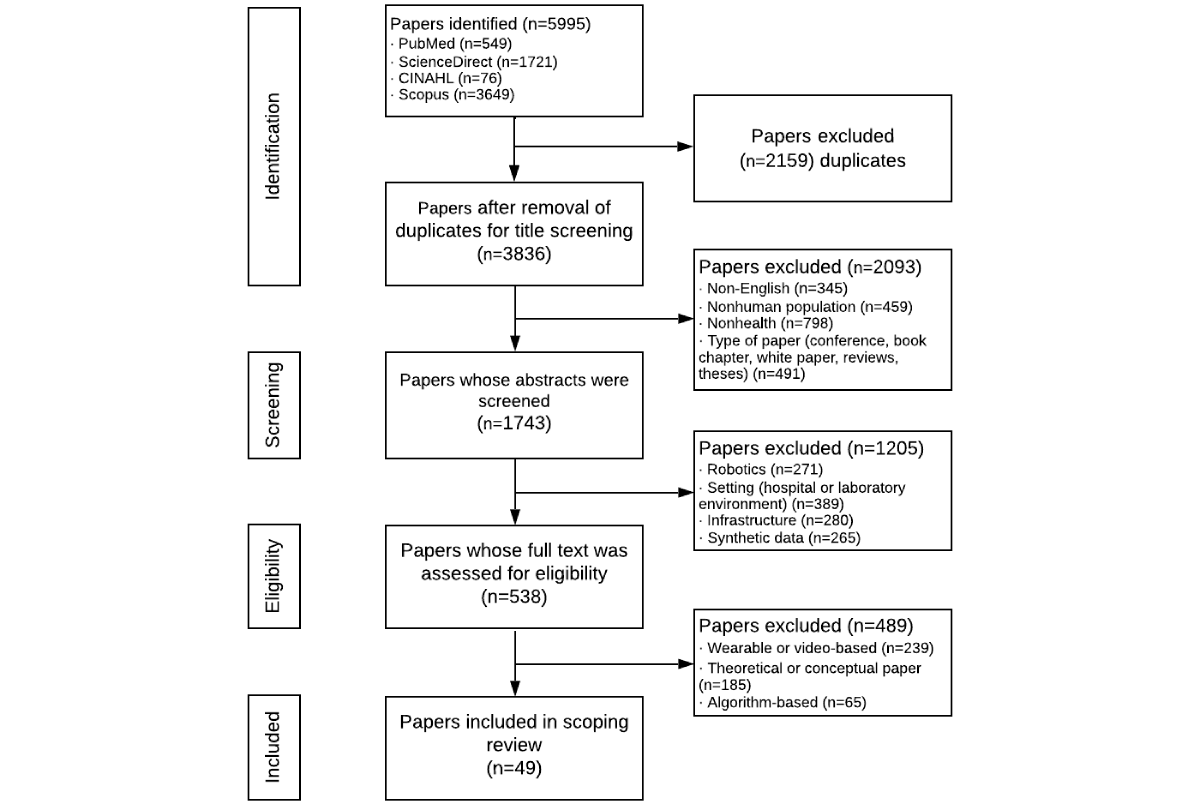
Systematic study selection using the PRISMA (Preferred Reporting Items for Systematic Reviews and Meta-Analyses) flowchart.

Papers were organized into Mendeley and Zotero reference managers. Following the removal of 2159 duplicate papers, 3836 papers remained for title screening (Figure 1). Paper selection was further refined by ensuring that paper titles contained one of our keywords as mentioned above. AO and KSS each reviewed half of the papers. Papers not in English and those not related to humans were excluded: papers related to animal, agricultural, or biology research were excluded. Further, conference papers, book chapters, white papers, reviews, and theses were removed. Following title screening, 1743 papers were selected for abstract review by AO and KSS in Mendeley. AO and KSS screened the abstracts to ensure that the papers focused on remote sensor technology and its application in a home setting. Papers that used synthetic data or described infrastructure architecture or were in hospital or laboratory settings were excluded. The remaining 538 papers proceeded to full-text screening and were transitioned to Zotero for file management due to software issues in Mendeley. Studies using wearables or video-based technologies, theoretical or conceptual papers, and algorithm-based technologies were removed. Both authors independently and unanimously agreed on the inclusion of 29 papers with an additional 97 papers with conflicting votes. These papers were discussed on a case-by-case basis until a unanimous decision was reached. Of the 97 papers that had conflicting votes, 20 papers were included in this review. Thus, 49 papers were found to be eligible for the final scoping review. The selected papers were saved in a database, and a master chart was built by AO and KSS to summarize the key information for subsequent analysis.

## Results

### Selection and Characterization of Studies on Smart Home Technologies

To gain an understanding of the types of smart home technologies being used and the information collected, we conducted a literature search across 4 databases (PubMed, Scopus, ScienceDirect, and CINAHL) between January 2008 and August 2021 by using the queries outlined in Table S1 of Multimedia Appendix 1. A total of 49 papers met the inclusion criteria for this scoping review (Table 1). Among the types of studies conducted, 31% (13/49) were pilot studies, 14% (7/49) were proof of concept, 12% (6/49) were algorithm evaluations, 10% (5/49) were proposals, 8% (4/49) were technical validations, 8% (4/49) were case studies, 6% (3/49) were method evaluations, 6% (3/49) were longitudinal studies, 4% (2/49) were platform evaluations, 2% (1/49) were randomized controlled trials, and 2% (1/49) were qualitative studies. When we examined the country of origin for each paper, we found that most of the studies were conducted in western societies, with 47% (23/49) of the papers originating from Europe and 35% (17/49) from North America. Few studies were conducted in Asia (6/49, 12%), Africa (2/49, 4%), and Oceania (1/49, 2%).

**Figure 2:**
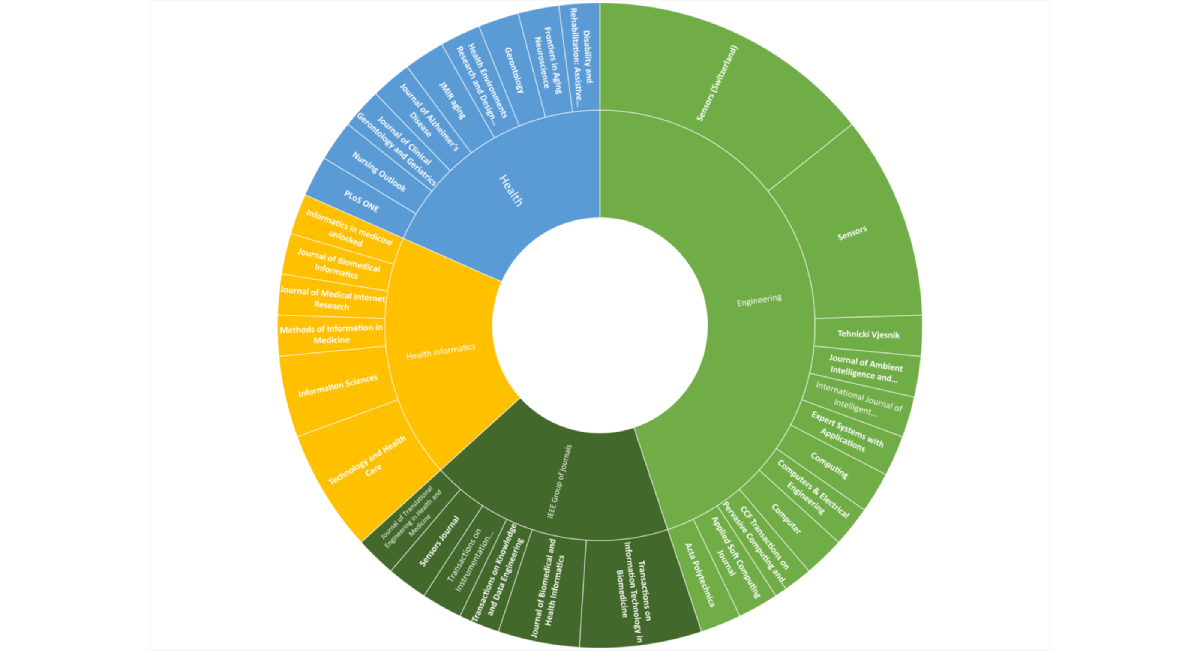
Journals of the published papers reviewed in this study.

We observed an increase in the number of publications in recent years: 71% (35/49) of the papers were published within the last 5 years (2015-2020), while only 29% (14/49) of the papers were published before 2015. All the studies were either directly or indirectly associated with academic institutions. When classified based on a publication’s domain, 64% (31/49) of the selected papers were published primarily in the fields of engineering and computer science, 18% (9/49) were published in biomedical engineering and health informatics journals, and 18% (9/49) were published in health-related journals (Figure 2 and Table S2 of Multimedia Appendix 1). 

**Table 1:** Profile of the selected studies by type and human participation.

Type of study, reference		Sample size	Demographic profile of the participants (age [years], male/female)	Participant health profile
**Pilot studies (n=13)**				
	Chen et al [14]	5	>45, 2 males, 3 females	Spinal cord injury, muscular dystrophy, multiple sclerosis, polio
	Bock et al [15]	11	>18	Healthy
	Fritz and Dermody [16]	10	>55	Chronic diseases
	Skubic et al [17]	34	>70	Chronic diseases
	Dawadi et al [18]	263	>18, 72 males, 191 females	Healthy
	Choi et al [19]	37	>65, 7 males, 30 females	Chronic diseases
	Clemente et al [20]	6	No data	No data
	Pigini et al [21]	32	No data	Healthy and cardiac conditions
	Monteriù et al [22]	13	>65	Healthy
	Grgurić et al [23]	13	>65	No data
	Dasios et al [24]	2	>70, 1 male, 1 female	Healthy
	Marcelino et al [25]	23	>30, 11 males, 12 females	Healthy
	Yu et al [26]	1	>65, 1 female	Chronic diseases
**Proof of concept (n=7)**				
	Kim et al [27]	20	>65	Depression
	Alberdi Aramendi et al [10]	29	>18	Healthy
	Hassan et al [28]	0	N/A^a^	N/A
	Shirali et al [29]	1	>65	No data
	Jung [30]	22	>60, 10 males, 12 females	No data
	Alsina-Pagès et al [31]	0	No data	N/A
	Mahmoud et al [32]	1	No data	Healthy
**Algorithm evaluation (n=6)**				
	Jakkula and Cook [33]	1	>18	Healthy
	Rashidi et al [34]	40	>18	Healthy
	Singla et al [35]	40	No data	Healthy
	Damodaran et al [36]	0	N/A	N/A
	Hamad et al [37]	19	No data	No data
	Enshaeifar et al [38]	12	No data	Dementia
**Proposals (n=5)**				
	Ros et al [39]	0	N/A	N/A
	Navarro et al [40]	0	N/A	N/A
	Gayathri et al [41]	0	N/A	N/A
	Kwon et al [42]	150	>60, 23 males, 127 females	Healthy
	Taiwo and Ezugwo [43]	0	N/A	N/A
**Technical validation (n=4)**				
	Mora et al [44]	0	N/A	N/A
	Bassoli et al [45]	0	N/A	N/A
	Schlebusch [46]	10	>18, 7 males, 3 females	Healthy
	Virone et al [47]	22	>45, 7 males, 15 females	Healthy
**Case studies (n=4)**				
	Sprint et al [12]	3	>70, 3 females	Lung cancer, insomnia, leg pain
	Lazarou et al [48]	4	>70, 1 male, 3 females	Amnestic, mild cognitive impairment, dementia
	Hercog et al [49]	1	>60, 1 female	Healthy
	Yang and Hsu [50]	0	N/A	N/A
**Method evaluation (n=3)**				
	Yao et al [51]	0	N/A	N/A
	Fleury et al [52]	13	>18	Healthy
	Fiorini et al [53]	17	>18	Healthy
**Longitudinal studies (n=3)**				
	Fritz et al [54]	11	>65	No data
	Austin et al [55]	16	>70, 3 males, 13 females	Healthy
	Lyons et al [56]	480	>70	No data
**Platform evaluation (n=2)**				
	Junnila et al [57]	2	>70, 1 male, 1 female	Healthy and hip surgery rehabilitation
	Lamprinakos et al [58]	207	>65	Frailty
**Randomized controlled trial (n=1)**				
	Mora et al [1]	78	>18, 69 males, 9 females	Healthy
**Qualitative study (n=1)**				
	Cahill et al [59]	200	No data	No data

^a^N/A: not applicable.

### Population Demographics

As it is common practice in computer science or engineering research to use simulated data for platform or algorithm evaluation, we first categorized the studies based on the source of their data. Approximately 78% (38/49) of the papers used data collected from human participants, and the remaining 22% (11/49) of the studies used simulated data (Table 1). The age of the participants ranged from 18 to 93 years. Of the 38 studies that utilized human participants, 63% (31/49) reported participant age, but only 33% (16/49) indicated the gender of the participants. Of those that did report gender, female participants were nearly 3 times more prevalent than male participants (425 females vs 145 males). Volunteer participants were typically students recruited from the researcher’s institution or patients from memory care units and assisted-living residents. Of the papers on human participants, 79% (30/49) reported the health status of the participants.

### Study Settings and Parameters

The 49 papers included in this review can be broadly divided into 2 groups: 41% (20/49) approached the use of IoT for health purposes and 59% (29/49) used IoT for technological validations. The primary research focus was recognizing human mobility patterns (Table 2; complete data in Table S3 of Multimedia Appendix 1). Study length ranged from a single day of data collection to 8 years. Data were collected primarily in real-world settings, including smart apartments or smart workplaces. One of the studies used simulated home environments [39]. If the study took place in an apartment, the number of rooms typically used was between 2 and 3. Typically, there was only a single occupant in the study location.

**Table 2:** Technical components of the selected studies with outcomes.

Type of study, reference			Primary focus		Outcome measure		Algorithm		Type of data
**Pilot study**									
	Chen et al [14], Dasios et al [24], Yu et al [26]	Independent living for the older population who may or may not have chronic diseases		Activity, fall detection, indoor motion		Statistical analysis of the machine learning algorithm		Binary sensors: motion, light, temperature, humidity,	
	Marcelino et al [25]	e-Service provision		Physical, medical, social interaction by audio-visual communication with service providers		Qualitative and quantitative data analysis		Interview questionnaire	
**Proof of concept**									
	Alberdi Aramendi et al [10], Kim et al [27], Hassan et al [28], Shirali et al [29], Jung [30], Alsina-Pagès et al [31], Mahmoud et al [32]	From 2013 to 2020, the proof of concept improved from synthetic data to real-world data, single individual to multi-individual, but the objectives more or less—the same activity recognition, anomaly detection, pattern recognition to improve the quality of life of older individuals		Motion or presence data		Binary sensor data, machine learning algorithm-support vector machine as the typical model with many of the studies; the recent study used the parallel activity log inference algorithm		Sensor data	
**Algorithm evaluation**									
	Jakkula and Cook [33], Rashidi et al [34], Singla et al [35], Damodaran et al [36], Hamad et al [37], Enshaeifar et al [38]	All the studies tried to recognize normal activity patterns and anomaly detection		Motion or presence data, device-free solutions based on radio signals like home Wi-Fi 802.11 channel state information		Machine learning and deep learning algorithms		Passive infrared sensors	
**Proposal**									
	Ros et al [39], Navarro et al [40], Gayathri et al [41], Kwon et al [42], Taiwo and Ezugwo [43]	Activity recognition of the individual		Mobility pattern recognition		Machine learning, deep learning algorithms		Binary sensor and acoustic sensor data	
**Technical validation**									
	Mora et al [44], Bassoli et al [45], Schlebusch [46], Virone et al [47]	Active assisted living monitoring, intelligent toilet seat, differentiate regular patterns, and identify abnormalities in household activities		Passive infrared sensors, magnetic contact, bed occupancy, chair occupancy, toilet presence, fridge sensor, electrocardiogram and bioimpedance spectroscopy measurements, behavioral monitoring by presence data		Behavior explanatory models, sensor profiles, multivariate habits clusters, R-peak detection, software for automatic measurement of circadian activity deviation/circadian activity rhythms		Motion sensor data, electrocardiogram, bioimpedance spectroscopy, passive infrared sensor	
**Case studies**									
	Sprint et al [12], Lazarou et al [48], Hercog et al [49], Yang and Hsu [50]	Behavior change detection, home monitoring system, activity recognition, effective active home automation solution based on open-source home automation software, and wireless, custom-developed, Wi-Fi–based hardware		Activity change, sleep, physical activity, and activities of daily living, automatic classification of activities of daily living, system functionality		CASAS^a^ middleware		Motion, light, temperature, door, motion, presence, utility usage sensors, passive infrared/current sensors	
**Method evalution**									
	Yao et al [51], Fleury et al [52], Fiorini et al [53]	Activity recognition		Automatic classification of activities of daily living		Support vector machine, unsupervised machine learning, rule-based reasoning method for activity recognition		Location, temperature, sound, postural transitions and walk periods, motion sensor, location, activity, motion	
**Longitudinal study**									
	Fritz et al [54], Austin et al [55], Lyons et al [56]	Remote monitoring of pain, loneliness		Recognize pain-associated behaviors		Machine learning algorithm, isolation forest (forest) anomaly detection algorithm, decision tree classifier, logistic regression classifier		Passive infrared–based sensor data, light, temperature, humidity	
**Platform evaluation**									
	Junnila et al [57], Lamprinakos et al [58]	Remote patient monitoring using home health or telehealth		Interoperability/adaptability, which can accommodate different types of sensors		Rule-based ontological framework, partial human monitoring is required		Passive infrared–based sensor data	
**Qualitative study**									
	Cahill et al [59]	Identify and validate the requirements for new technology enabling resident wellness and person-centered care delivery in a residential care environment		State of environment and state of care delivery, state of resident		Qualitative data analysis and machine learning algorithm		Sensor and interview data	
**Randomized controlled trial (secondary data analysis)**									
	Mora et al [1]	Internet of Things–based home monitoring for older patients with stroke		Behavioral aspects-bed/rest patterns, toilet usage, room presence, and many others		Regression framework and anomaly detection, unsupervised clustering techniques		Sensor data	

^a^CASAS: Center for Advanced Studies in Adaptive Systems

### Data Collection and Analysis

To determine which smart home technologies were being used, sensors were grouped into 16 main categories (Table 3): utilization of space (bed and chair occupancy, toilet, fridge, kitchen, or GPS), human vitals (blood pressure, electrocardiography, blood glucose, heart rate, or respiratory rate), and environmental sensors (light, air temperature, humidity, sound, airflow, smoke, carbon monoxide, gas, or flooding). Nearly 62% (30/49) of the studies used passive infrared sensors to report on motion detection. As motion detectors and object presence sensors primarily record binary (yes/no) data, it was unsurprising that this data type was the most reported in the studies examined. Quantitative data were reported in many papers. Audiovisual (sound, light), vital indicators (heart rate, respiratory, blood glucose, body temperature), and environmental conditions (room temperature, humidity) typically record quantitative data. Finally, several papers reported spatiotemporal data typical of GPS sensors.

As smart home data collection produces large quantities of data, data management software is frequently employed. Examining the papers, we found SQL [34,35,56,57] and MYSQL [24,25,55] were frequently used to organize the data. MATLAB and Python were used for data analysis and visualization by nearly all the studies. Various statistical methods were used for data analysis, including descriptive statistics, model building, machine learning, and deep learning. Descriptive statistics were primarily used to describe the demographic characteristics of the study participants, whereas multidomain approaches [52], longitudinal linear mixed-effect regression [55], and out-of-sample cross-validation methods [55] were used for statistical models.

As 41% (20/49) of the papers reported the use of machine learning algorithms, we sought to determine which algorithms were more commonly employed. Clustering in 5 studies [1,28,30,34,53] and Hidden Markov Model in 4 studies [23,30,34,39] were the most used in data analysis to identify a regular pattern and predict future patterns. The other algorithms used in the studies were decision tree emerging pattern [11,25,27], clustering conditional random field [37,51], context-aware reasoning [28,42], fuzzy logic [41,49], k-nearest neighbors [10,51], logistic regression classifier [51,55], AdaBoost [10], Bayes network [27], boosting model using ensemble [42], circadian activity rhythms [47], multi-Hidden Markov Model [34], multiple regression model [42], multivariate habits cluster [44], ontological modelling [41], software for automatic measurement of circadian activity deviation [47], and support vector machines [52].

Nearly 14% (7/49) of the papers used deep learning methods, which included artificial neural networks [40], activity recognition using the discontinuous varied-order sequential model [34], latent trajectory models [56], longitudinal linear mixed-effect regression recurrent neural networks [55], open pass neural networks [60], recurrent neural networks [32], and multilayer perceptron [10]. One study used mixed methods and included a thematic analysis of the quantitative data [25]. Another study used the activity discovery method [34], and yet another conducted qualitative data analysis by using a mixed methods approach [25]. Some studies used induction algorithms, behavioral monitoring systems, rapid iterative testing and evaluation [15], or QRS recognition [57] for electrocardiography.

**Table 3:** Types of sensors, data characteristics, and their association with health.

Sensor type	Data type	Health indicator/proxy
Motion: passive infrared sensors, radiofrequency identification, magnetic switches	Any movement within the room, door movement	Physical activity/speed/quality of physical health/sleep
Presence	Any movement within the room, indoor movement	Physical activity/gait speed/quality of physical health/sleep
Temperature	Temperature of room, temperature of stove/oven	Body temperature, health quality/activity-sleep/awake/sedentary
Light	Luminosity (lux)	Sleep/active
Sound/microphone	Noise	Sleep/active
Humidity	Indoor environment	Indoor environment
Biosensors	Fall detection	Activity/alert
Plug sensors	Appliance use: television, fridge, kitchen appliance, medicine dispenser	Activity
Body position sensors	Activity	Activity
Carbon monoxide	Indoor environment	Indoor environment
Flooding sensors	Water use/consumption	Indoor environment
Gas sensors	Use of gas in the kitchen	Indoor environment
Smoke detector	Indoor environment	Indoor environment
Pressure sensor/smart tiles/pressure pad	Bed movement, gait speed, chair movement	Sleep time/quality
Electrocardiogram patch	Heart health	Heart health
Airflow sensors	Room environment	Indoor environment
SpO_2_	Oxygen saturation of blood	Heart health/lung health
Blood pressure	Heart health	Heart health
Heart rate	Heart health	Heart health
Respiratory rate	Lung health	Lung health
Blood glucose sensors	General health	Diabetes
Smart weighing scale	Body weight	Weight
Pedometer	Walking	Physical activity
Contact sensors	Usage of a phone book, cooking pot, medicine container	Activity analysis
GPS	Location	Location
Wi-Fi signal	Indoor activity	Location
Smart seismic sensor	Floor vibration	Activity analysis, including fall

### Outcome Measures

All the studies reported that IoT improved the quality of care, increased participants’ sense of comfort, enabled early detection, and increased participants’ understanding of the impact of health events on overall health. The health indicators specifically measured through smart home technologies included fall detection [24], functional health decline/improvement [10], high-level activities of daily living/instrumental activities of daily living [34,35,48,50,59,61-63], leisure services [59], loneliness [55], medical services [17,21,30,64], patient health status [17,21,30,64], perception [58], physical activity [48], sedentary behaviors [24,62], medication adherence [62], movement patterns [29], sequence of gestures [61], sleep [12,48,56], eating habits [10,24,57,62], situational awareness [30], social engagement [56], time spent outside the home [55], and overall well-being [24].

### Limitations and Challenges in the Studies

To gain insight into future research needs in the field of smart home technologies, we extracted information pertaining to the challenges and limitations self-reported by researchers. In the 49 studies, the biggest challenge faced by the researchers was differentiating between multiple participants in a single space. The second challenge identified was the lack of technology interoperability and the ability to scale up. The third challenge identified was linked to data security and privacy. The additional challenges identified by the researchers included calibration of the sensors, cost of technology and data management, data streaming and integration, data velocity, data volume, difficulty differentiating activities, generalization of activities, and demographic discrepancies (data collected from young volunteers, while algorithms were designed for the older population). Heterogeneity, installation of the sensors, lack of patient motivation, large numbers of nodes, limited data bandwidth, limited indoor activities, malfunctioning sensors, privacy, sample size, security, service quality, user acceptance, and varying levels of data accuracy were also noted as challenges.

## Discussion

### Key Findings

Existing reviews on the application of smart home technology for health care are limited [2,3]. If at all present, they focus on a very specific specialty within health care, such as geriatric care [65], dementia [66,67], fall prevention [68], or pregnancy [69]. This scoping review aims to address this knowledge gap by elucidating how smart home technologies are being used for health care within the home and community. An extensive database search revealed 49 peer-reviewed publications, which met our inclusion criteria. A wide variety of sensors were used to meet the differing needs in each study. Passive infrared sensors, which report on motion detection, were the most studied smart home technology for health and report primarily binary data. Multiple studies quantified measurable health indicators (eg, heart rate, blood pressure, sleep, physical activity). Reported data were mostly organized using SQL or MYSQL. As expected, diverse data analyses and statistical methods, including machine learning and deep learning, were applied to big data analysis. Of note, although some studies were performed in home settings, none were unobtrusive or zero effort. There were often disruptions to daily routines or participants were required to log activities [70].

We recognize that there are several limitations to our study and that potentially relevant publications may have been overlooked due to the constraints in our search queries and inclusion criteria. As smart home technologies are often developed by the technology industry, not all work is likely published in peer-reviewed journals. Furthermore, our use of the query term “smart home” may have excluded relevant research settings in a community or an institution. For the purposes of this scoping review, database searches were conducted in August 2021. Due to the rapid nature of this field of research, new insights may have emerged since the initial search.

### Defining a “Smart Home” for Health Care

During the preparation of this scoping review, we noted that the health care research papers lacked a concrete definition for a smart home. Based on the available evidence and the identified gaps, we propose the following definition for a smart home for health care.

A smart home for health care can be defined as a home equipped with smart sensors using Bluetooth, Wi-Fi, or similar technology, not restricted to IoT, to automate, regulate, and monitor home occupants’ physical health, mental health, and environments within the home. The focus must be on convenience, safety, and improvement of one’s quality of life, to address the needs of the individual, caregivers, and health professionals.

### Sociodemographic Inequalities

The studies included in this review were predominantly performed in western societies. This bias could be due to our requirement that studies should be published in English. However, it is known that high-income nations dominate the field of smart home technology. This could be due to several factors. First, western countries are trending toward an aging population, and thus, the interest in assisted living technologies is higher [71]. Second, low- and middle-income countries are focused on reducing mortality and morbidity related to infectious diseases; therefore, their resources are not focused on the needs of an aging population [72-74]. To address global health and knowledge inequalities, researchers and funding bodies must ensure that low- and middle-income countries have the resources to benefit from health technologies. Future research should prioritize including study participants in nonwestern societies.

Computer science or engineering research often use simulated data due to budget, staffing, and time constraints. Traditional technical training does not consider health outcomes and overlooks the social determinants of health. Without health care experts as part of the research team, many are unaware of the importance of reporting the demographic characteristics of human study participants. This was reflected in our scoping review, as many of the included studies failed to report this information. Of those that did report demographics, we found that female participants were more prevalent, being nearly 3 times more likely to have been studied than male participants. This was unexpected, given that research is typically dominated by male participants [75,76]. Some potential reasons for this variance could be that women live longer [77], are more likely to live in assisted care units [78], are more likely to participate in studies [79], or have altruistic considerations [80]. Moreover, the use of simulated data despite the availability of actual data highlights the need for better access to high-quality data.

### The Intersection of Health and Technology

Smart home technology is a rapidly growing interdisciplinary field at the intersection of health, information technology, and engineering [81]. Yet, our scoping review highlighted a strong bias toward publication within primarily engineering and information technology journals. Many of the papers included in this scoping review contained highly technical language, tools, and databases. However, the primary audience is the health care field. Although we acknowledge that much of the technology is in its early stages, with research focused on technical challenges (data handling, analysis, storage, security, and privacy), this finding highlights a lack of collaboration between health and technical fields. Future work must address this gap—fostering interdisciplinary research teams with a broad spectrum of skills and domain knowledge experts. The involvement of health professionals in smart home technology research will ensure that these tools are relevant and bolster their successful implementation.

### Technological Challenges

Interoperability was a commonly noted challenge faced by researchers. Technology is constantly being upgraded and improved with new products continually hitting the market. As diverse companies compete to create the latest technology, interoperability becomes an issue. Because there are no standardized guidelines, companies develop their own unique protocols and architectures for handling data, which contribute to incompatibility across the IoT landscape. The result is a jungle of systems that are confusing and intimidating to navigate for many non–tech-savvy individuals. One must subscribe to a single system that may not meet all their needs, grapple with the inconvenience of systems that do not communicate seamlessly, or implement third-party software or hardware to bridge the gap. There is a need to continue to develop solutions that allow these systems to integrate and communicate with one another. Similarly, the other 2 challenges faced by the researchers were differentiating individuals within a multiparticipant household and data security and privacy. Health care technology brings a new layer of complexity due to risks associated with personally identified data, health data, privacy, data rights, and ethical considerations [82].

### Data Quality

Some of the studies [18,27,55] examined here had insufficient data quality to make their research findings relevant in the health care field. In many cases, the number of study participants was minimal and lacked demographic information. The quality of many of the sensors used in a home setting is lesser than that of the instruments used in a clinical setting, often diminishing the value of the data. Additionally, some of the technologies were not diagnostic tools at all because the health indicators were not quantifiable (video or audio). Other health conditions such as loneliness or mental health cannot be quantified and thus must be measured through the integration of multiple proxy indicators. The challenges of data integration will likely be addressed with continued improvements in artificial intelligence. Here, we have highlighted the existing research on the application of smart home technology to improve health and revealed multiple gaps in our knowledge. The IoT has ushered in a period of ultraconnectivity [83], converting commercial, off-the-shelf sensors like smart Wi-Fi thermostats and wearable devices into vital sources of health data. With the collaborative efforts of technology experts and health care professionals, we have the potential to leverage these data to improve physical and mental health.

### Conclusion

Smart home technology has the potential to improve the quality of life by monitoring health indicators in vulnerable persons. Despite their potential, there is still a lack of large-scale utilization of these technologies for health care. A scoping review of the existing literature enabled us to identify the types of sensors and the data being explored. The trends and knowledge gaps identified here will invite new progress in remote patient monitoring in public health. This kind of a care system can support and complement medical interventions to improve population health.

## Appendix

Multimedia Appendix 1 Supplementary data.

## Abbreviations

IoT: Internet of Things
