# Supplementary material for: Health Monitoring Using Smart Home Technologies: Scoping Review
Source: JMIR Mhealth Uhealth. 2023 Apr 13;11:e37347. doi: 10.2196/37347 (PMC10141305; doi:10.2196/37347)
Supplement: Multimedia Appendix 1 [file mhealth_v11i1e37347_app1.docx]

# **Table S1.** Search strategy in detail

| **Database** | **Number of Articles** | **Query** |
| --- | --- | --- |
| PubMed | 549 | (((((((((("smart home" AND Health* AND monitor*)))))) OR ((((((AAL AND Health* AND monitor*))))))) OR (((((("ambient assisted living" AND Health* AND monitor*)))))) OR (((((("active assisted living" AND Health* AND monitor*))))) |
| Science Direct | 1,721 | ("smart home*" AND health* AND monitor*) OR (AAL AND health* AND monitor*) OR ("ambient assisted living" AND Health* AND monitor*) OR ("active assisted living" AND health*) |
| Scopus | 3649 | Total |
|  | 509 | "Smart home" health* monitor* |
|  | 538 | AAL AND health* AND monitor |
|  | 2573 | "ambient assisted living" AND Health* AND monitor* |
|  | 29 | "active assisted living" AND Health* AND monitor* |
| CINAHL | 76 | Total |
|  | 13 | "Smart home" health* monitor* |
|  | 2 | AAL AND health* AND monitor |
|  | 6 | "ambient assisted living" AND Health* AND monitor* |
|  | 55 | "active assisted living" AND Health* AND monitor* |
| **All database** | **5995** |  |

Note: Label: behavio*, individual, population

Not limited to geographic location

Limiters: English, human, 2007-2020

Exclude: Not English

Include: research articles, review articles

Searched on Aug 22, 2021, at 10:00 am

# **Table S2.** Domain and Journal of publication of the selected articles

| **Domain** | **Name of Journal** | **Number of Publications** |
| --- | --- | --- |
| Engineering | Sensors (Switzerland) | 7 |
| Engineering | Sensors | 5 |
| IEEE Group of journals | Transactions on Information Technology in Biomedicine | 3 |
| IEEE Group of journals | Journal of Biomedical and Health Informatics | 2 |
| Engineering | Acta Polytechnica | 1 |
| Engineering | Applied Soft Computing Journal | 1 |
| Engineering | CCF Transactions on Pervasive Computing and Interaction | 1 |
| Engineering | Computer | 1 |
| Engineering | Computers & Electrical Engineering | 1 |
| Engineering | Computing | 1 |
| Engineering | Expert Systems with Applications | 1 |
| Engineering | International Journal of Intelligent Information Technologies | 1 |
| Engineering | Journal of Ambient Intelligence and Humanized Computing | 1 |
| Engineering | Tehnicki Vjesnik | 1 |
| IEEE Group of journals | Journal of Translational Engineering in Health and Medicine | 1 |
| IEEE Group of journals | Sensors Journal | 1 |
| IEEE Group of journals | Transactions on Instrumentation and Measurement | 1 |
| IEEE Group of journals | Transactions on Knowledge and Data Engineering | 1 |
| Health informatics | Technology and Health Care | 3 |
| Health informatics | Information Sciences | 2 |
| Health informatics | Informatics in medicine unlocked | 1 |
| Health informatics | Journal of Biomedical Informatics | 1 |
| Health informatics | Journal of Medical Internet Research | 1 |
| Health informatics | Methods of Information in Medicine | 1 |
| Health | Disability and Rehabilitation: Assistive Technology | 1 |
| Health | Frontiers in Aging Neuroscience | 1 |
| Health | Gerontology | 1 |
| Health | Health Environments Research and Design Journal | 1 |
| Health | JMIR ageing | 1 |
| Health | Journal of Alzheimer's Disease | 1 |
| Health | Journal of Clinical Gerontology and Geriatrics | 1 |
| Health | Nursing Outlook | 1 |
| Health | PLoS ONE | 1 |

**Table S3.** Technical components of the selected studies with outcomes.

| Type of study, reference | | Primary focus | Outcome measure | Algorithm | Type of data | Key finding | Challenge |
| --- | --- | --- | --- | --- | --- | --- | --- |
| **Pilot study** | | | | | | | |
|  | Chen et al [14], Dasios et al [24], Yu et al [26] | Independent living for the older population who may or may not have chronic diseases | Activity, fall detection, indoor motion | Statistical analysis of the machine learning algorithm | Binary sensors- motion, light, temperature, humidity, | - All the studies said they were successful with their objectives. - Monitoring of physical, social, and cognitive health of healthy persons or those with chronic diseases is possible using existing mechanisms. - Multiple types of sensors can be integrated to build a central service, where monitoring/early identification of abnormal events can be possible. | Differentiate individuals and false alarm |
|  | Marcelino et al [25] | e-Service provision | Physical, medical, social interaction by audio-visual communication with service providers | Qualitative and quantitative data analysis | Interview questionnaire | Everyone was able to perform the tasks without any help or intervention. | Scalability |
| Proof of concept | Alberdi et al [10], Kim et al [27], Hassan et al [28], Shirali et al [29], Jung [30], Alsina-Pagès et al [31], Mahmoud et al [32] | From 2013 to 2020, the proof of concept improved from synthetic data to real-world data, single individual to multi-individual, but the objectives more or less—the same- activity recognition, anomaly detection, pattern recognition to improve the quality of life of older individuals | Motion or presence data | Binary sensor data- Machine learning algorithm-support vector machine as the typical model with many of the studies. The recent study used the Parallel Activity Log Inference Algorithm. | Sensor data | Successfully proved their work, and all of them were working. | Real-world data with noise, multi-person discrimination |
|  |  |  |  |  |  |  |  |
|  |  |  |  |  |  |  |  |
|  |  |  |  |  |  |  |  |
|  |  |  |  |  |  |  |  |
|  |  |  |  |  |  |  |  |
|  |  |  |  |  |  |  |  |
|  |  |  |  |  |  |  |  |
|  |  |  |  |  |  |  |  |
|  |  |  |  |  |  |  |  |
|  |  |  |  |  |  |  |  |
|  |  |  |  |  |  |  |  |
|  |  |  |  |  |  |  |  |
| Algorithm evaluation | Cook and Jakkula [33], Rashidi et al [34], Singla et al [35], Damodaran et al [36], Hamad et al [37], Enshaeifar et al [38] | All the studies tried to recognize normal activity patterns and anomaly detection | Motion or presence data, device-free solutions based on radio signals like (home Wi-Fi 802.11 channel state information) | Machine learning and deep learning algorithms | Passive infrared sensors | - With time, the accuracy of the algorithms increased. - Precision and sensitivity increased significantly when using preceding and partial oncoming sensor activations compared to when using only preceding sensor activation. | The real-time capabilities have become a key challenge in activity recognition to offer a tool that meets real-world conditions. |
|  |  |  |  |  |  |  |  |
|  |  |  |  |  |  |  |  |
| Proposal | Ros et al [39], Navarro et al [40], Gayathri et al [41], Kwon et al [42], Taiwo and Ezugwo [43] | Activity recognition of the individual | Mobility pattern recognition | Machine learning, deep learning algorithms | Binary sensors and Acoustic sensors data | Better performance in abnormality detection than that of the existing systems, monitoring of the patient in real time, which reduces hospital costs and exposure of patients to highly contagious diseases like COVID-19 |  |
| Technical validation | Mora et al [44], Bassoli et al [45], Schlebusch [46], Virone et al [47] | Active assisted living monitoring, Intelligent toilet seat, differentiate regular patterns and identify abnormalities in household activities | Passive infrared sensors, magnetic contact, bed occupancy, chair occupancy, toilet presence, fridge sensor, electrocardiogram and bioimpedance spectroscopy measurements, behavioral monitoring by presence data | Behavior explanatory models, Sensor profiles, and multivariate habits clusters, R-peak detection, Software for Automatic Measurement of Circadian Activity Deviation/Circadian Activity Rhythms | Motion sensor data, electrocardiogram, and bioimpedance spectroscopy, passive infrared sensor | - Convincing proof-of-concept test, demonstrating the ability to elicit expressive indications from indirect, continuous monitoring, fully autonomous. - The intelligent toilet has a high potential to increase the quality of medical care for older adults living at home by keeping track of important health and nutrition parameters. - The system can model behavioral patterns and detect deviations that may be consistent with disease onset. | These test methods are not diagnostic modes.  Need to test the same with a real-world scenario. Only indoor activity can be monitored and discriminating multiple individuals in a single room. |
|  |  |  |  |  |  |  |  |
|  |  |  |  |  |  |  |  |
|  |  |  |  |  |  |  |  |
|  |  |  |  |  |  |  |  |
|  |  |  |  |  |  |  |  |
|  |  |  |  |  |  |  |  |
|  |  |  |  |  |  |  |  |
|  |  |  |  |  |  |  |  |
|  |  |  |  |  |  |  |  |
|  |  |  |  |  |  |  |  |
|  |  |  |  |  |  |  |  |
|  |  |  |  |  |  |  |  |
|  |  |  |  |  |  |  |  |
|  |  |  |  |  |  |  |  |
| Case studies | Sprint et al [12], Lazarou et al [48], Hercog et al [49], Yang and Hsu [50] | Behavior change detection, home monitoring system, Activity recognition effective active home automation solution based on open-source home automation software and wireless, custom developed, Wi-Fi–based hardware. | Activity change, sleep, physical activity, and activities of daily living, Automatic classification of activities of daily living, System functionality | CASAS middleware | Motion. Light temperature, door, motion, presence, utility usage sensors, passive infrared/current sensors | - Smart-home and machine-learning technologies can be used to understand the behavioral impacts of health events. - Successful measurement of daily activities | Recalling retrospective data, sensors can be updated, heterogeneity in data collection. Data were from the household member of the author. Unable to distinguish visitor/cohabitant, Case study without human participant involvement |
|  |  |  |  |  |  |  |  |
|  |  |  |  |  |  |  |  |
|  |  |  |  |  |  |  |  |
|  |  |  |  |  |  |  |  |
|  |  |  |  |  |  |  |  |
|  |  |  |  |  |  |  |  |
| Method evaluative | Yao et al [51], Fleury et al [52], Fiorini et al [53] | Activity recognition | Automatic classification of activities of daily living | Support vector machine, Unsupervised machine learning, Rule-based reasoning method for activity recognition | Location, temperature, sound, postural transitions and walk periods, Motion sensor, location, activity, motion | - The cross-validation test gave preliminary results with a classification rate of 75% for a polynomial kernel and 86% for a Gaussian kernel with an adapted parameter. - The night-time period was found to be adequate in capturing baseline behavior patterns. - The minimal number of days needed to establish baseline user behavior patterns is 55. - The recognition accuracy outperformed well-known methods. | Real-world location, multi-occupancy noise |
| Longitudinal study | Fritz et al [54], Austin et al [55], Lyons et al [56] | Remote monitoring of pain, loneliness | Recognize pain-associated behaviors | Machine learning algorithm, isolation forest (forest) anomaly detection algorithm, Decision tree classifier, logistic regression classifier | Passive infrared based sensors data, light temperature humidity | Effective for detecting clinically relevant pain-related behavior or changes relevant to those behaviors, Prediction of loneliness possible using sensor data. | Data security and privacy, distinguishing individuals within the home |
| Platform evaluation | Junnila et al [57], Lamprinakos et al [58] | Remote patient monitoring using home health or telehealth | Interoperability/adaptability which can accommodate different types of sensors | Rule-based ontological framework, Partial human monitoring is required. | Passive infrared-based sensors data | - 2010 study was with two patients, whereas the 2015 study tested more than 200 frail patients. - Using these patient-reported enhanced senses of security. - Positive outcome toward combined monitoring early detection of health events. | To discriminate the presence of external persons in the patient’s indoor environment, such as visitors, to reassure the correctness of the collected activity information, always in an as unobtrusive as possible manner. |
|  |  |  |  |  |  |  |  |
|  |  |  |  |  |  |  |  |
|  |  |  |  |  |  |  |  |
| Qualitative study | Cahill et al [59] | Identify and validate the requirements for new technology enabling resident wellness and person-centered care delivery in a residential care environment | State of environment and state of care delivery, state of resident | Qualitative data analysis and machine learning algorithm | Sensor and interview data | Participants have control over a particular aspect of their life and sense of comfort | Additional research is required with older adults with diverse age-related physical, sensory, and cognitive challenges. |
|  |  |  |  |  |  |  |  |
|  |  |  |  |  |  |  |  |
| Randomized controlled trial (Secondary data analysis) | Mora et al [1] | Internet of things based home monitoring for older patients with stroke | Behavioral aspects-bed/rests patterns, toilet usage, room presence and many others | Regression framework and anomaly detection, unsupervised clustering techniques | Sensor data | The study was successful with the objectives. | Scaling up of these findings to broader systems |
|  |  |  |  |  |  |  |  |
